# BTBD10 inhibits glioma tumorigenesis by downregulating cyclin D1 and p-Akt

**DOI:** 10.1515/biol-2022-0103

**Published:** 2022-08-10

**Authors:** Yu Liu, Sen Li, Ruoping Chen, Juxiang Chen, Bo Xiao, Yicheng Lu, Jiangang Liu

**Affiliations:** Department of Neurosurgery, Shanghai Children’s Hospital, School of Medicine, Shanghai Jiao Tong University, Shanghai, 200000, China; Department of Neurosurgery, Shanghai Changzheng Hospital, Shanghai, 200000, China

**Keywords:** glioma, BTBD10, proliferation, apoptosis, cyclin D1, Akt

## Abstract

The aim of this study was to investigate the role of BTBD10 in glioma tumorigenesis. The mRNA and protein levels of BTBD10 in 52 glioma tissues and eight normal brain tissues were determined using reverse transcription polymerase chain reaction (RT-PCR) and western blot analysis, respectively. U251 human glioblastoma cells were infected with BTBD10-expressing or control lentiviruses. Cell growth was evaluated using the methyl thiazolyl tetrazolium (MTT) assay. Cell apoptosis and cell cycle distribution were analyzed using flow cytometry. Cyclin D1 and p-Akt levels were determined using western blot analysis. The results showed that BTBD10 mRNA and protein levels were significantly lower in glioma tissues than in normal brain tissues. Additionally, BTBD10 levels were significantly lower in high-grade gliomas than in low-grade tumors. Compared with control cells, U251 cells overexpressing BTBD10 exhibited decreased cell proliferation, increased cell accumulation at the G0/G1 phase, increased cell apoptosis, and decreased levels of cyclin D1 and p-Akt. These findings show that BTBD10 is downregulated in human glioma tissue and that BTBD10 expression negatively correlates with the pathological grade of the tumor. Furthermore, BTBD10 overexpression inhibits proliferation, induces G0/G1 arrest, and promotes apoptosis in human glioblastoma cells by downregulating cyclin D1- and Akt-dependent signaling pathways.

## 1 Introduction

Gliomas comprise about 30% of all tumors of the adult central nervous system and 80% of all malignant brain tumors [1,2]. The current mainstay of treatment for glioma is the combined use of surgery, radiotherapy, and chemotherapy. However, malignant gliomas are often difficult or impossible to resect completely because they grow diffusely, infiltrate brain tissue, and have poorly defined margins [3]. In addition, the blood–brain barrier limits the delivery of drugs to the brain and thereby limits the efficacy of chemotherapy [4]. Consequently, malignant glioma is associated with a high recurrence rate and poor prognosis. Understanding the mechanisms involved in the pathogenesis of glioma is key to developing novel therapeutic agents for this tumor.

The BTB (BR-C, ttk, and bab) or POZ (Pox virus and zinc finger) [5] domain is an evolutionarily conserved structural domain found primarily in zinc finger proteins. The BTB/POZ domain has been shown to interact with components of histone deacetylase co-repressor complexes to repress gene transcription [6,7,8,9]. In addition, the BTB/POZ domain has been implicated in cytoskeleton organization, development, and carcinogenesis [10,11,12,13]. In 2004, a cDNA microarray analysis identified a novel cDNA that was downregulated in human glioma tissues [14]. This cDNA was found to encode a novel human BTB domain-containing protein, which was named BTBD10. The *BTBD10* gene showed a ubiquitous expression pattern, and the highest level of expression was found in the brain, testis, and small intestine. Additionally, the BTBD10 protein was detected mainly in the nucleus [14], implicating a functional role for the protein in transcriptional regulation. Interestingly, BTBD10 was not differentially expressed in hepatocellular carcinoma, ovarian cancer, or lung cancer [14], which suggests that BTBD10 may be specifically linked to glioma.

In this study, we examined the expression of BTBD10 in glioma and normal brain tissue obtained from patients who had undergone surgery. Furthermore, we evaluated the relationship between tumoral BTBD10 expression and clinicopathologic characteristics (including the pathological grade and type of glioma and the age and gender of the patient). We also investigated the effects of BTBD10 overexpression on the growth and apoptosis of a human glioblastoma cell line and explored the signaling pathways that mediated these effects.

## 2 Materials and methods

### 2.1 Human tissue samples

Tumor tissues were collected from 52 patients with glioma (29 males and 23 females) who had undergone brain surgery at Shanghai Changzheng Hospital between 2005 and 2008. The patients were 5–80 years old (average age, 43.1 years old) and had not received radiotherapy, chemotherapy, or immunotherapy prior to surgery. The specimens were examined by an experienced histopathologist and graded according to the 2000 World Health Organization criteria. The grade of the glioma was grade I in 6 patients (4 cases of fibrillary astrocytoma and 2 cases of ependymoma), grade II in 17 patients (6 cases of diffuse astrocytoma, 1 case of gemistocytic astrocytoma, 1 case of pleomorphic xanthoastrocytoma, 5 cases of oligodendroglioma, 2 cases of oligoastrocytoma and 2 cases of ependymoma), grade III in 13 patients (7 cases of anaplastic astrocytoma, 3 cases of anaplastic oligodendroglioma, and 3 cases of ependymoma), and grade IV in 16 patients (15 cases of glioblastoma multiforme and 1 case of gliosarcoma). Normal brain tissues used as controls were collected from eight patients with intracranial brain injury who underwent decompression surgery at the same hospital between 2005 and 2008. The control tissues were examined by a histopathologist to exclude the presence of any tumors. All tissue specimens were rinsed with physiological saline, and any parts that showed signs of bleeding, necrosis, or electrosurgical trauma were removed. The samples were subsequently frozen and stored in liquid nitrogen until analysis. The institutional review board of Shanghai Changzheng Hospital approved this study, and all study participants provided informed consent for their stored, unused tissue samples to be used in research studies.


**Informed consent:** Informed consent has been obtained from all individuals included in this study.
**Ethical approval:** The research related to human use has complied with all the relevant national regulations, and institutional policies and in accordance with the tenets of the Helsinki Declaration, and has been approved by the authors’ institutional review board or equivalent committee.

### 2.2 Reverse transcription-polymerase chain reaction (RT-PCR)

Total RNA was extracted using Trizol reagent (Invitrogen, USA), and cDNA was synthesized using avian myeloblastosis virus reverse transcriptase (Promega, USA) in accordance with the manufacturer’s instructions. PCR was performed on a RealPlex4 Real-time PCR system (Eppendorf, Germany) using SYBR Green Supermix (Takara Bio Inc., Japan). The following primers were synthesized at Sangon Biotech (China): BTBD10, 5′-AGCAGTGCTGGGAACAGCAGCAG-3′ (forward) and 5′-TATATTCCGAGCTCCTT-3′ (reverse); 18 S rRNA, 5′-CAGCCACCCGAGATTGAGCA-3′ (forward) and 5′-TAGTAGCGACGGGCGGTGTG-3′ (reverse); cyclin D1, 5′-AGGAGAACAAACAGATCA-3′ (forward) and 5′-TAGGACAGGAAGTTGTTG-3′ (reverse); and glyceraldehyde 3-phosphate dehydrogenase (GAPDH), 5′-CCACCCATGGCAAATTCCATGGCA-3′ (forward) and 5′-TCTAGACGGCAGGTCAGGTCCACC-3′ (reverse). Relative mRNA expression was calculated using the 2^−ΔΔCT^ method. The expression of BTBD10 was normalized to that of 18 S rRNA, and the expression of cyclin D1 was normalized to that of GAPDH.

### 2.3 Western blot analysis

The tissues and cells were lysed in radioimmunoprecipitation assay buffer and centrifuged at 13,000×*g* for 10 min at 4°C. The supernatants were collected, and their protein concentrations were determined using the Lowry method. Proteins were separated on 10% sodium dodecyl sulfate-polyacrylamide gel electrophoresis and transferred to polyvinylidene difluoride membranes (Millipore, USA). The membranes were blocked with 5% skim milk in Tris-buffered saline with Tween 20 for 3 h at 4°C. Then, the membranes were incubated with mouse anti-human BTBD10 polyclonal antibody (1:500; Abnova, Taiwan), mouse anti-human cyclin D1 polyclonal antibody (1:500; Santa Cruz Biotechnology, USA), or mouse anti-human phosphorylated Akt (p-Akt) polyclonal antibody (1:500, Santa Cruz Biotechnology) overnight at 4°C. Subsequently, the membranes were incubated with horseradish peroxidase-conjugated secondary antibody (Santa Cruz Biotechnology) for 1 h at room temperature. Protein bands were detected with an enhanced chemiluminescence reagent (Santa Cruz Biotechnology).

### 2.4 Cell culture

U251 human glioblastoma cells were obtained from the Shanghai Institute of Biochemistry and Cell Biology, Chinese Academy of Science. The cells were maintained in Dulbecco’s Modified Eagle Medium (Gibco, USA) supplemented with 10% fetal bovine serum (Gibco) at 37°C in a humidified atmosphere containing 5% CO_2_.

### 2.5 Lentiviral infection of U251 cells

The coding sequence of human BTBD10 was amplified by PCR using the following primers: 5′-CCGGGTACCGGAATTCCGCCACCATGGCAGGACGGCCTC-3′ (forward; the EcoRI recognition sequence is underlined) and 5′-CCTTGTAGTCGCTAGCCAGCATTGGATTCTGTGCATC-3′ (reverse; the NheI recognition sequence is underlined). The amplification product was inserted into EcoRI- and NheI-linearized pLV-UbC-GFP-3FLAG lentiviral vector (SunBio, China) and co-transfected with pCD/NL-BH*DDD packaging plasmids (Addgene, USA) and p-LTR-G envelope plasmids (Addgene) using Trans-EZ reagent (SunBio) into 293 T cells to generate Lenti-BTBD10. An empty vector (Lenti-EV) was used as the control. U251 cells were plated in six-well plates (2 × 10^5^ cells/well) and incubated for 72 h with Lenti-BTBD10 or Lenti-EV at a multiplicity of infection of 50 in the presence of 8 μg/mL polybrene. Subsequently, the cells were harvested, and the mRNA and protein levels of BTBD10 were determined using RT-PCR and western blot analysis, respectively.

### 2.6 Cell proliferation

Cell growth was evaluated using the MTT assay. In brief, cells in the logarithmic growth phase were seeded in 96-well plates at a density of 1 × 10^4^ cells/well and cultured for up to 5 days. Cell viability was determined on days 1, 2, 3, 4, and 5 using the MTT assay.

### 2.7 Cell apoptosis

Cells were harvested, fixed in 70% ethanol for 12 h at 4°C, and double-stained with propidium iodide (PI) and annexin V-fluorescein isothiocyanate (FITC). The cells were subsequently analyzed using a FACSAria flow cytometer (Becton Dickinson, USA). Quadrant plots were used to determine the level of cell apoptosis.

### 2.8 Cell cycle analysis

Cell cycle analysis was performed using flow cytometry. In brief, cells were harvested, fixed in 70% ethanol for 12 h at 4°C, and stained with PI in the presence of 50 μg/mL RNase A for 30 min at 37°C in the dark. The DNA content was determined using a FACSAria flow cytometer.

### 2.9 Statistical analysis

The data were analyzed using SPSS 15.0 software (SPSS Inc., USA). All results are presented as the mean ± standard deviation (SD). Grade I and grade II tumors were categorized as low-grade gliomas (*n* = 23), and grade III and grade IV tumors were classified as high-grade gliomas (*n* = 29). Inter-group comparisons were made using a one-way analysis of variance (for data that passed the homogeneity of variance test) or the Kruskal–Wallis test (for data that violated the inhomogeneity of variance test). Differences between the two groups were evaluated using the Student–Newman–Keuls test. The relationship between the mRNA expression of BTBD10 and the protein expression of BTBD10 in human tissues was evaluated using Pearson correlation analysis. All experiments were performed in triplicate. A *P*-value less than 0.05 was deemed statistically significant.

## 3 Results

### 3.1 BTBD10 expression was downregulated in human glioma tissue and negatively correlated with disease progression

**Figure 1:**
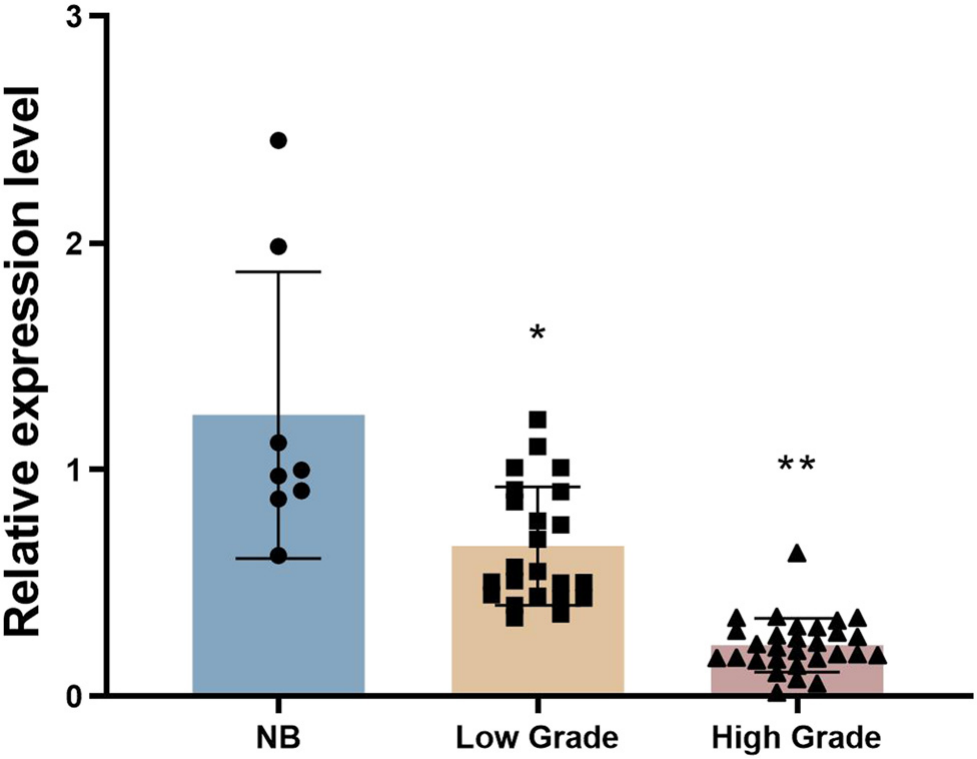
The mRNA expression of BTBD10 in normal brain (NB, *n* = 8), low-grade glioma (low grade, *n* = 23), and high-grade glioma (high grade, *n* = 29) tissues evaluated using RT-PCR. NB, non-tumor brain tissues. The data are presented as the mean ± SD. *, *P* < 0.05; **, *P* < 0.01.

**Figure 2:**
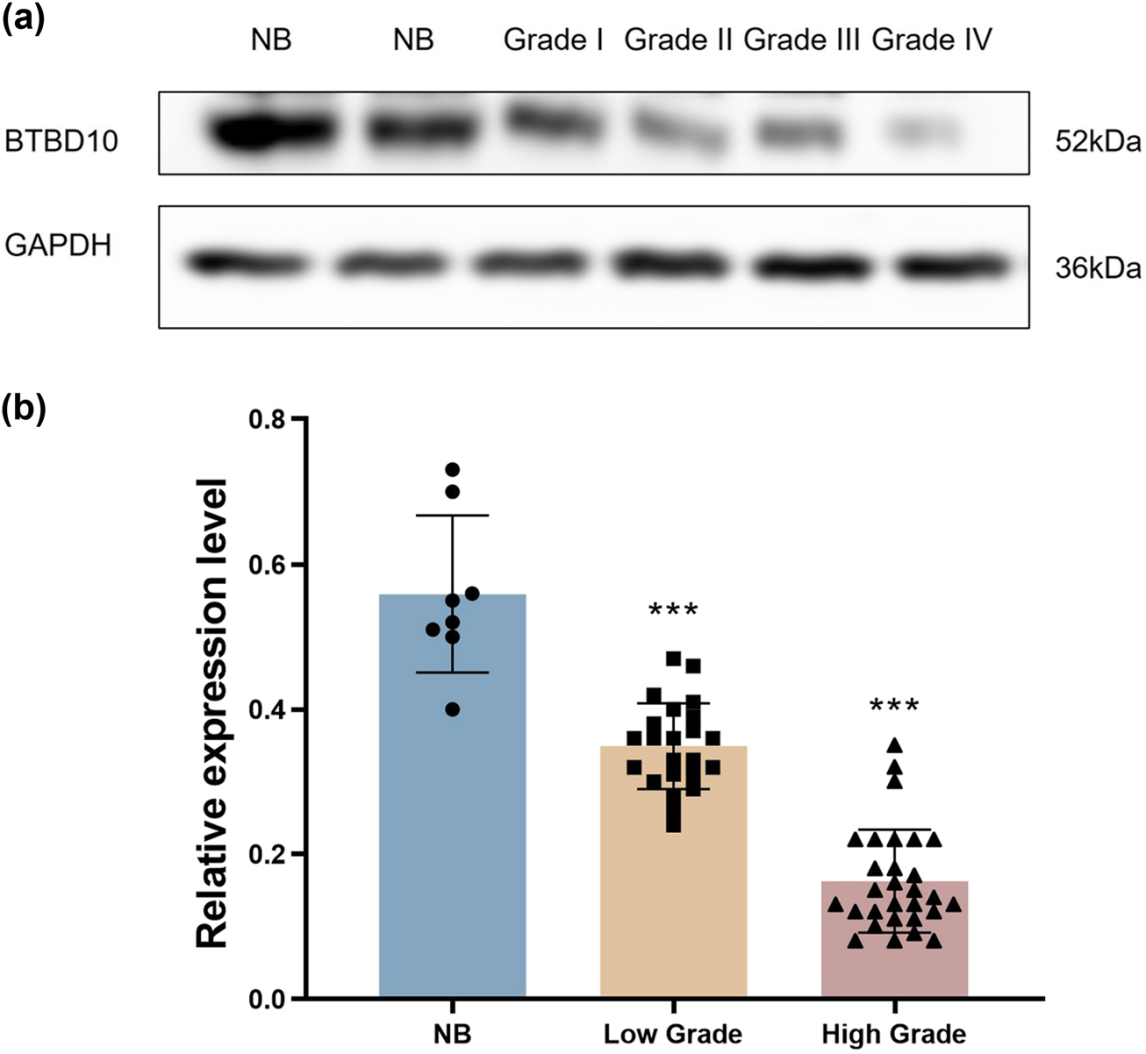
The protein expression of BTBD10 in normal brain (NB, *n* = 8), low-grade glioma (low grade, *n* = 23), and high-grade glioma (high grade, *n* = 29) tissues evaluated using western blot analysis. (a) Images showing representative gels; (b) quantified protein levels. The data are presented as the mean ± SD. NB, non-tumor brain tissues.***, *P* < 0.01 for all pairwise comparisons between groups.

**Figure 3:**
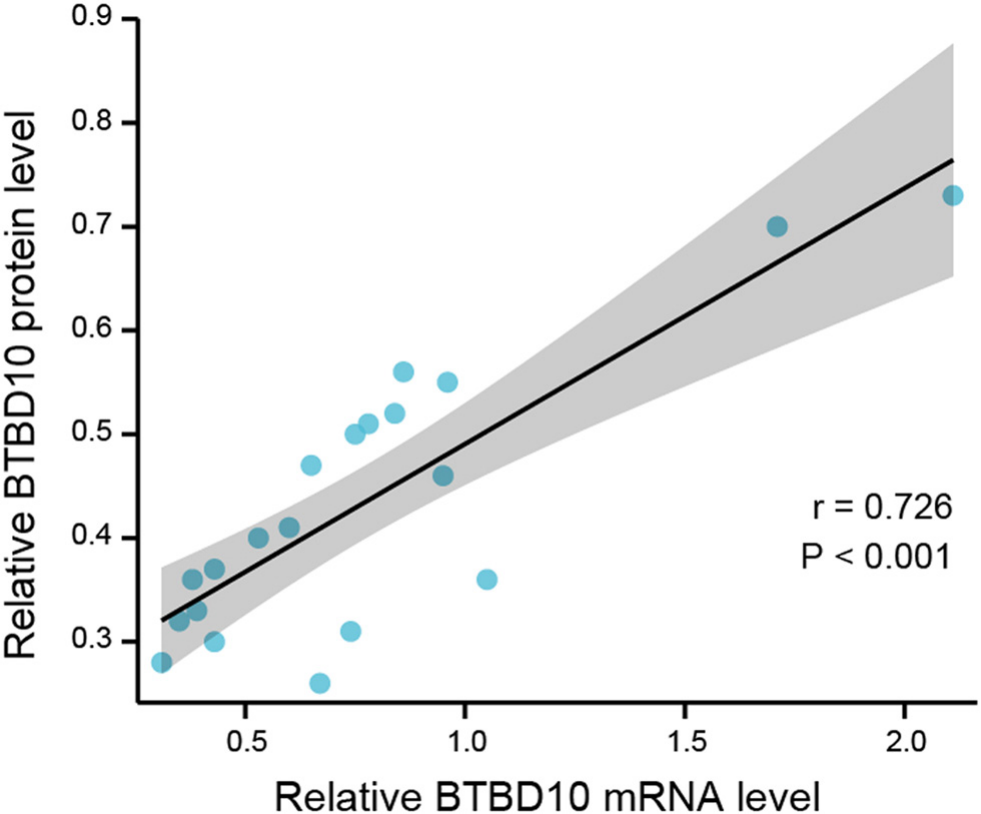
Pearson correlation analysis of the relationship between BTBD10 mRNA expression and BTBD10 protein expression (*r* = 0.726, *P* < 0.001).

The workflow of the present study is shown in the Supplemental Figure. The RT-PCR data revealed that *BTBD10* mRNA expression was highest in normal brain tissue (1.07 ± 0.54), intermediate in low-grade glioma tissue (0.57 ± 0.23) and lowest in high-grade glioma tissue (0.19 ± 0.10; *P* = 0.037 and *P* = 0.003, respectively; Figure 1). Similarly, BTBD10 protein expression was highest in normal brain tissue (0.58 ± 0.11), intermediate in low-grade glioma tissue (0.35 ± 0.06), and lowest in high-grade glioma tissue (0.16 ± 0.07; *P* < 0.01 between any two of the three groups, Figure 2). Pearson correlation analysis revealed a significant positive association between *BTBD10* mRNA expression and BTBD10 protein expression in the tissues examined (*r* = 0.726, *P* < 0.001; Figure 3). Collectively, the above data indicate that BTBD10 expression is downregulated in human glioma tissue and negatively correlated with disease progression. However, BTBD10 expression in glioma tissue was not significantly associated with the age or gender of the patient or the pathological type of the disease (*P* > 0.05, Tables 1 and 2).

**Table 1:** The relationship between tumoral BTBD10 mRNA expression and the clinicopathologic characteristics of the 52 patients with glioma

Clinicopathologic characteristic		Number	*P*
Gender	Male	31	0.564
	Female	21	
Age	<50 years	35	0.199
	>50 years	17	
Pathological type	Astrocytic tumor	37	0.423
	Oligodendroglial tumor	8	
	Ependymal tumor	7	
Pathological grade	Low grade	23	<0.001
	High grade	29	

**Table 2:** The relationship between tumoral BTBD10 protein expression and the clinicopathologic characteristics of the 52 patients with glioma

Clinicopathologic characteristic		Number	*P*
Gender	Male	31	0.722
	Female	21	
Age	<50 years	35	0.187
	>50 years	17	
Pathological type	Astrocytic tumor	37	0.623
	Oligodendroglial tumor	8	
	Ependymal tumors	7	
Pathological grade	Low grade	23	<0.001
	High grade	29	

### 3.2 Overexpression of BTBD10 inhibited the proliferation of human glioblastoma cells

**Figure 4:**
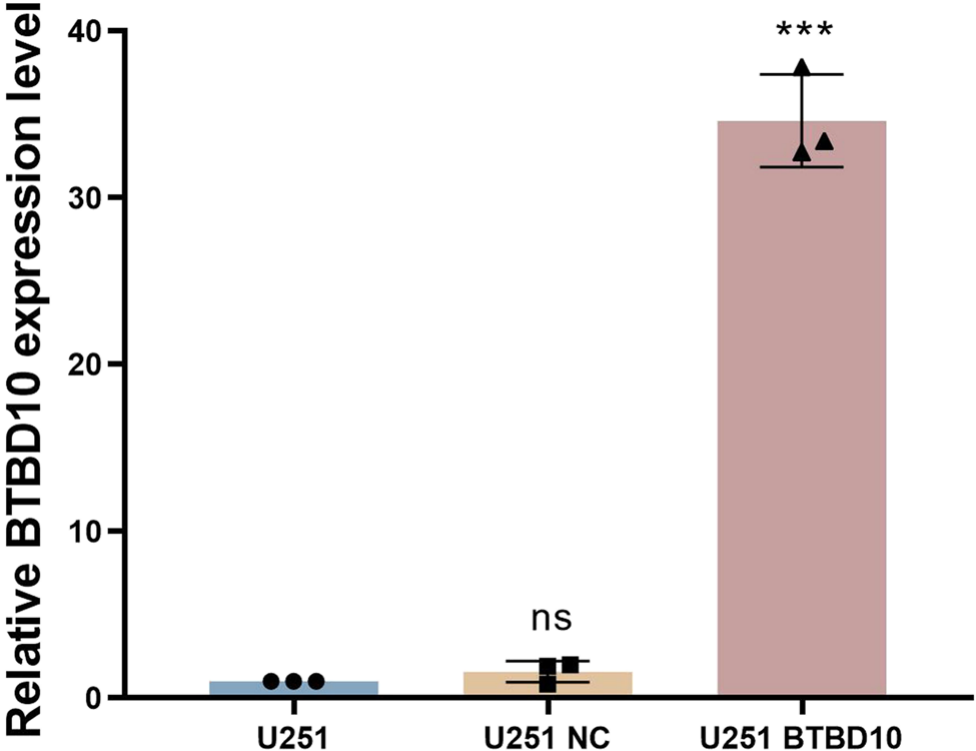
U251 cells were infected with Lenti-BTBD10 or Lenti-EV for 72 h. BTBD10 mRNA levels in uninfected (U251), Lenti-EV-infected (U251 NC), and Lenti-BTBD10-infected (U251 BTBD10) cells were determined using RT-PCR. The data are presented as the mean ± SD (*n* = 3 per group). ***, *P* < 0.01; ns, not significant.

**Figure 5:**
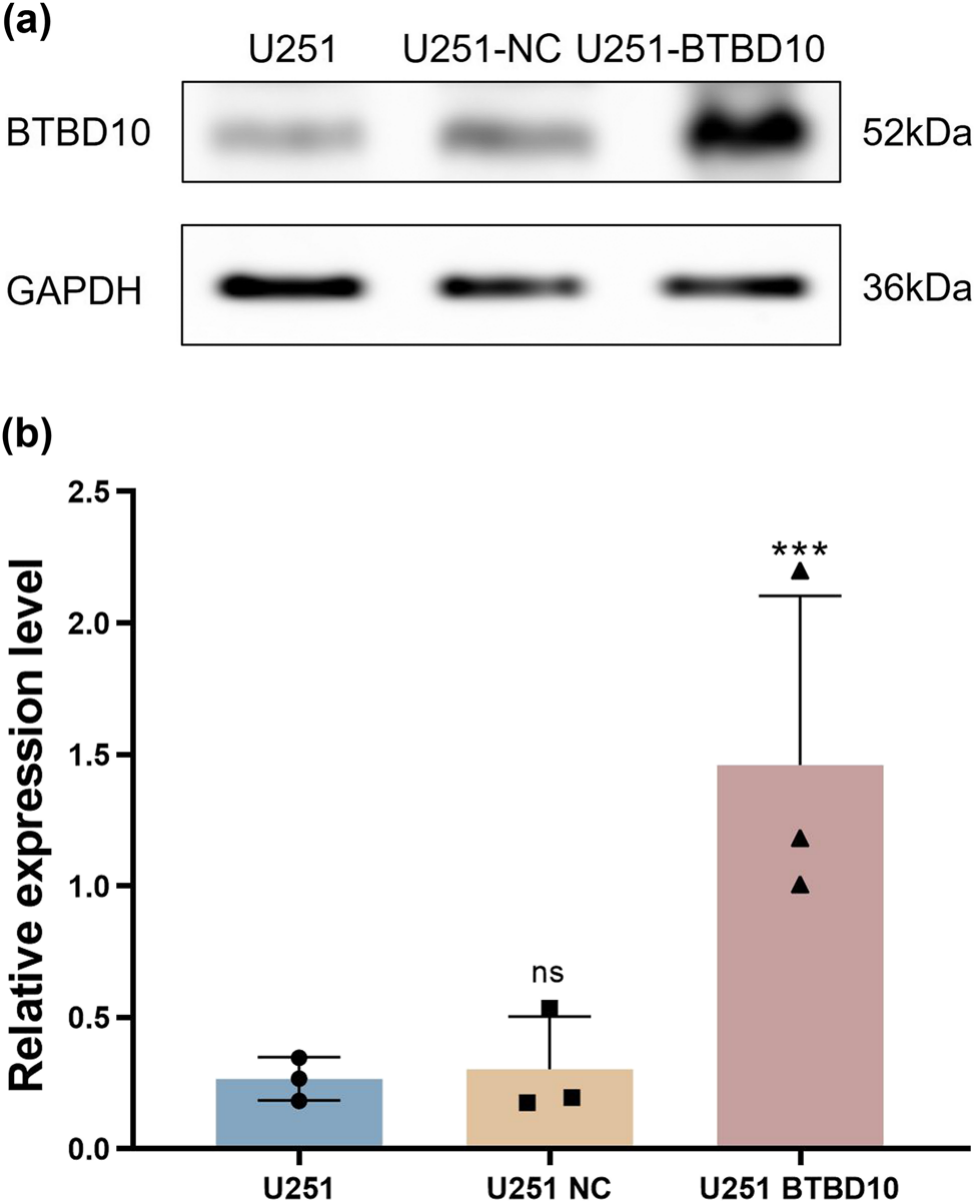
U251 cells were infected with Lenti-BTBD10 or Lenti-EV for 72 h. BTBD10 protein levels in uninfected (U251), Lenti-EV-infected (U251 NC), and Lenti-BTBD10-infected (U251 BTBD10) cells were determined using western blot analysis. (a) Images of representative gels; (b) quantified protein levels. The data are presented as the mean ± SD (*n* = 3 per group). ***, *P* < 0.01; ns, not significant.

**Figure 6:**
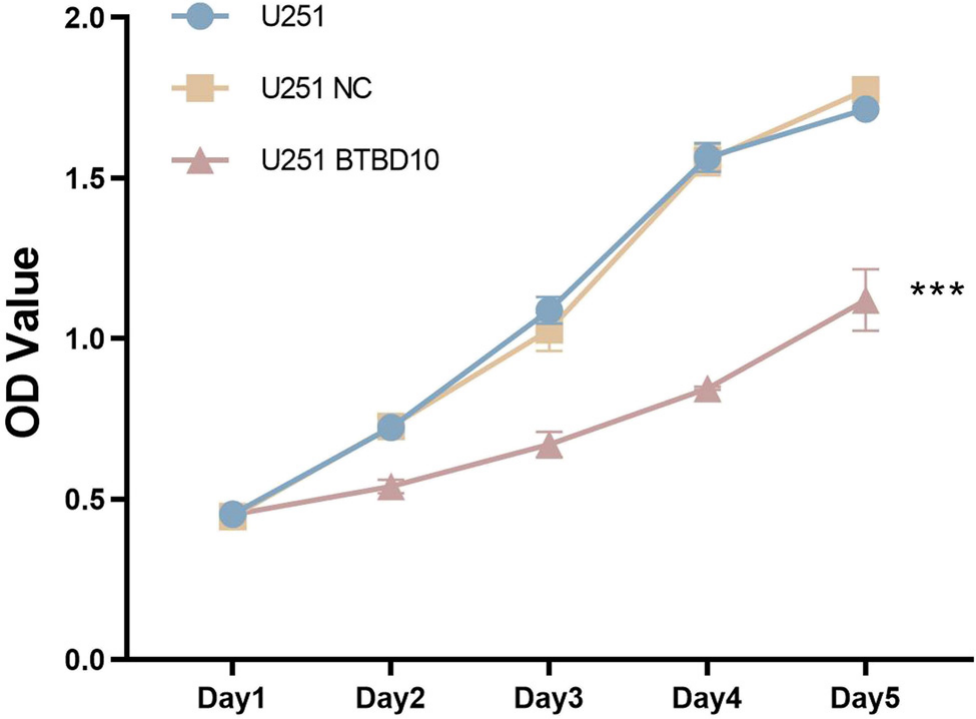
Growth curves of uninfected (U251), Lenti-EV-infected (U251 NC), and Lenti-BTBD10-infected (U251 BTBD10) cells obtained using the MTT assay. The data are presented as the mean ± SD (*n* = 3 per group). ***, *P* < 0.01.

U251 human glioblastoma cells were infected with Lenti-BTBD10 or Lenti-EV for 72 h. RT-PCR (Figure 4) and western blot (Figure 5) analyses confirmed that BTBD10 was overexpressed in Lenti-BTBD10-infected cells but not Lenti-EV-infected cells. Methyl thiazolyl tetrazolium (MTT) assays revealed that cell proliferation between day 2 and day 5 was significantly lower for Lenti-BTBD10-infected cells than for uninfected or Lenti-EV-infected cells (*P* < 0.01, Figure 6). The growth rate of Lenti-EV-infected cells was similar to that of uninfected cells between days 2 and 5 (*P* > 0.05, Figure 6).

### 3.3 Overexpression of BTBD10 promoted the apoptosis of human glioblastoma cells

**Figure 7:**
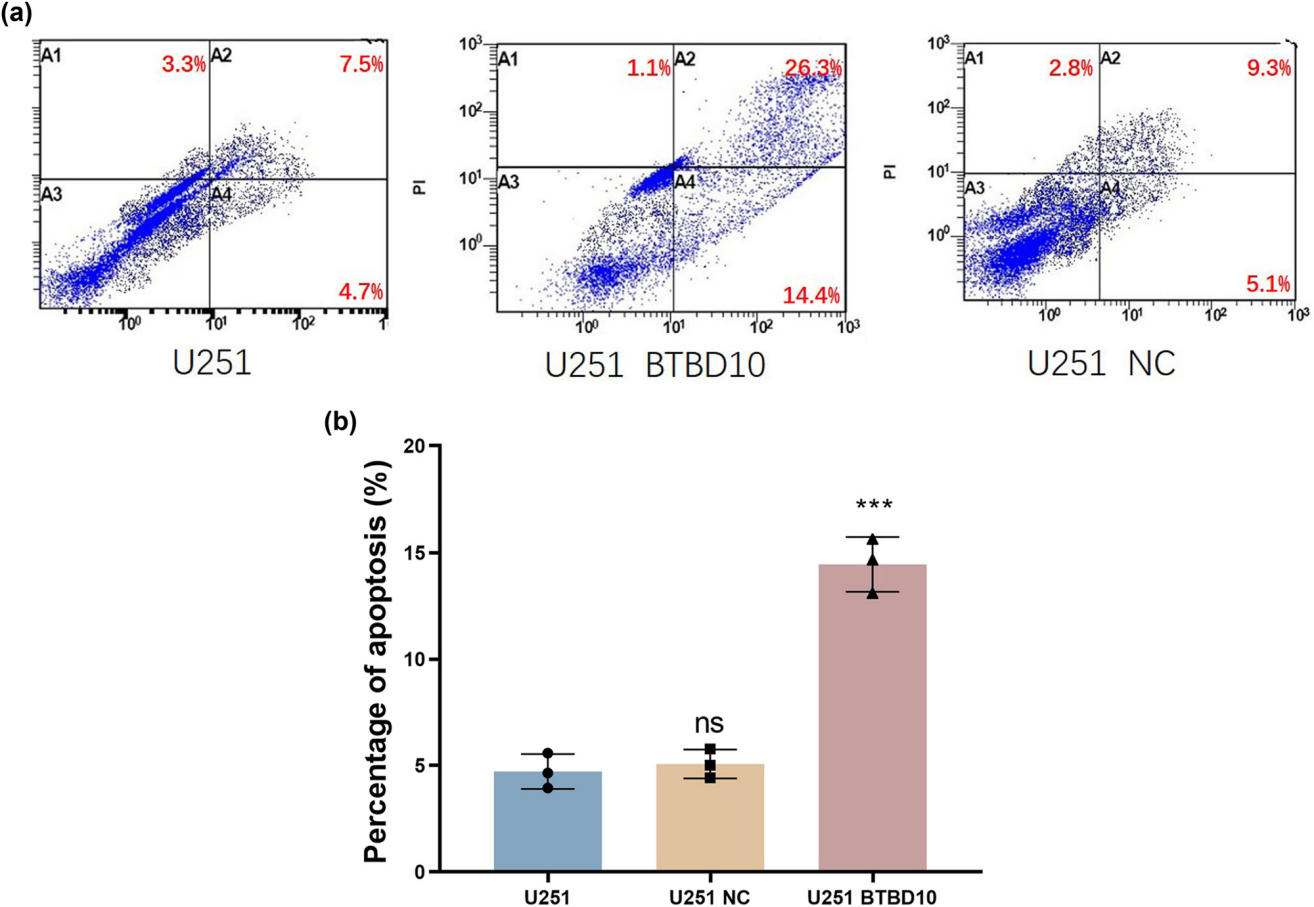
The apoptosis of uninfected (U251), Lenti-EV-infected (U251 NC), and Lenti-BTBD10-infected (U251 BTBD10) cells was evaluated using flow cytometry. (a) Representative quadrant diagrams; (b) quantified rates of apoptosis. The data are presented as the mean ± SD (*n* = 3 per group). ***, *P* < 0.01; ns, not significant.

Flow cytometric analyses of cells double-stained with annexin V-FITC, and PI revealed that the rate of apoptosis was significantly higher for Lenti-BTBD10-infected U251 cells (14.4%) than for uninfected (4.7%) or Lenti-EV-infected (5.1%) cells (*P* < 0.05, Figure 7). There was no significant difference in the rate of apoptosis between Lenti-EV-infected and uninfected cells (*P* > 0.05, Figure 7).

### 3.4 Overexpression of BTBD10 induced the arrest of human glioblastoma cells in the G0/G1 phase

**Figure 8:**
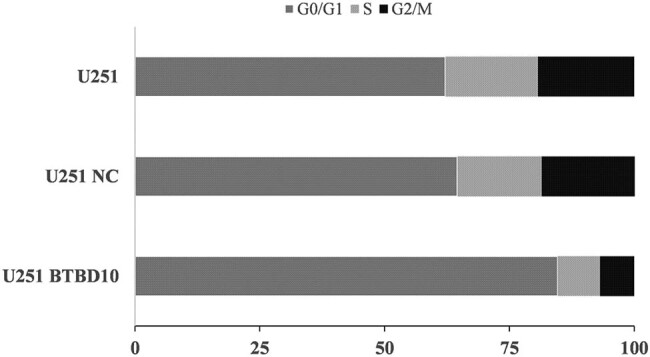
Cell cycle analysis using flow cytometry. U251, uninfected U251 cells; U251 NC, Lenti-EV-infected U251 cells; U251 BTBD10, Lenti-BTBD10-infected U251 cells. Mean values are shown (*n* = 3 per group).

Flow cytometry-based cell cycle analyses were carried out to investigate the mechanisms underlying the inhibitory effects of BTBD10 overexpression on U251 cell proliferation. The proportion of cells in the G0/G1 phase was significantly higher in Lenti-BTBD10-infected cells (84.7%) than in uninfected (62.2%) or Lenti-EV-infected (64.5%) cells (*P* < 0.01, Figure 8). No significant differences in cell cycle distribution were detected between uninfected and Lenti-EV-infected U251 cells (*P* > 0.05, Figure 8). The above data indicate that overexpression of BTBD10 inhibited the proliferation of U251 cells by inducing arrest in the G0/G1 phase.

### 3.5 BTBD10 overexpression downregulated cyclin D1 and p-Akt in human glioblastoma cells

**Figure 9:**
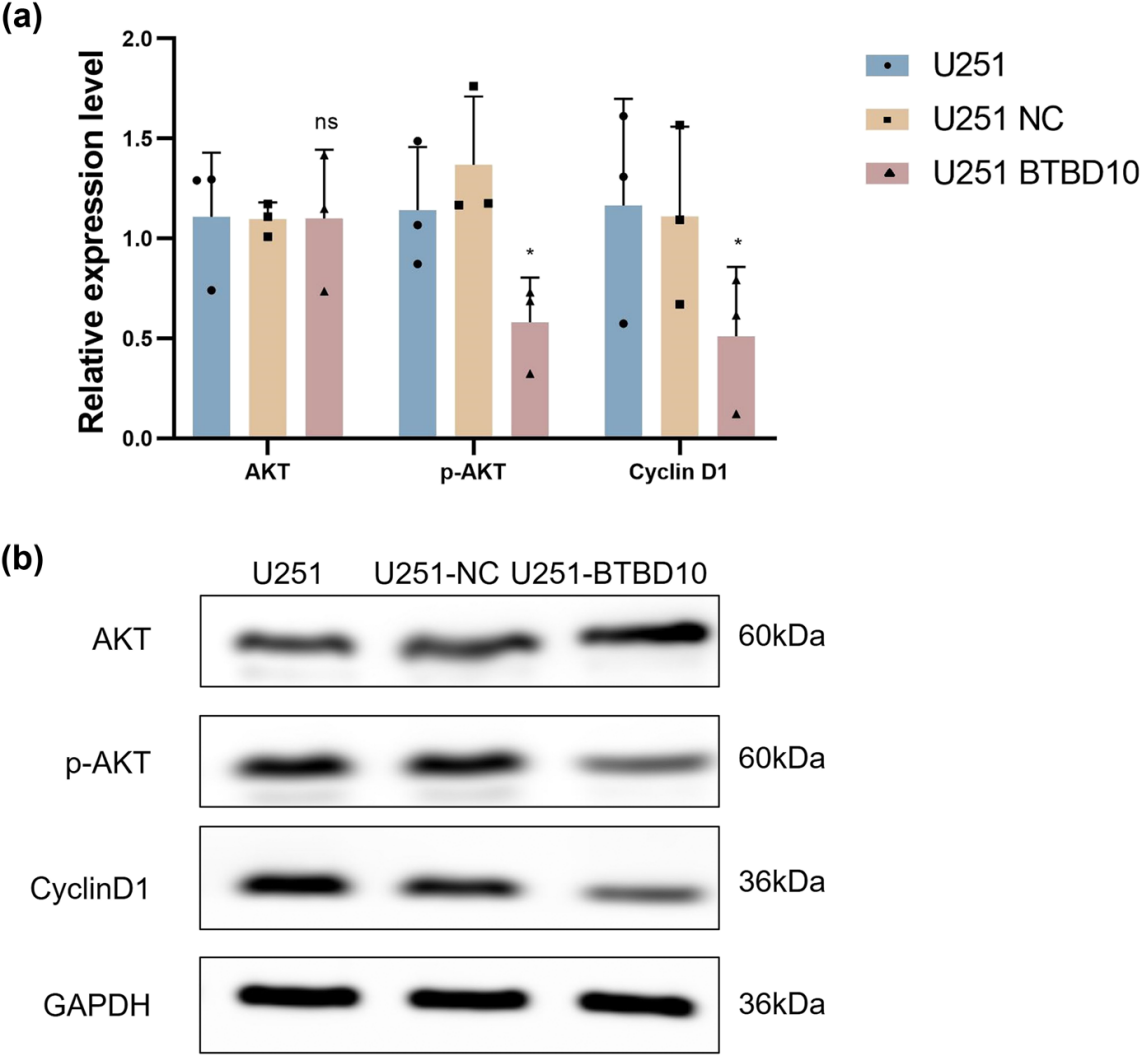
Analysis of cyclin D1 and p-Akt levels in uninfected (U251), Lenti-EV-infected (U251 NC), and Lenti-BTBD10-infected (U251 BTBD10) cells. (a) Cyclin D1, Akt, and p-Akt protein levels determined using western blot analysis. (b) Quantified protein levels are shown. The data are presented as the mean ± SD (*n* = 3 per group). *, *P* < 0.05; ns, not significant.

To explore the signaling pathways that might be involved in the effects of BTBD10 overexpression in U251 cells, we examined the protein levels of cyclin D1, Akt, and p-Akt using western blot analysis. Compared with uninfected or Lenti-EV-infected cells, Lenti-BTBD10-infected cells showed significantly decreased levels of cyclin D1 and p-Akt, while no significant difference was observed in the level of Akt (Figure 9). Thus, the anti-tumorigenic effects of BTBD10 overexpression in glioma cells were likely mediated, at least in part, by cyclin D1- and Akt-dependent signaling pathways.

## 4 Discussion

BTBD10 was first identified by Chen et al. in 2004 [11], who reported that BTBD10 contains a BTB/POZ domain and is downregulated in glioma but not in other types of tumor. Although these findings implicated BTBD10 in the pathogenesis of glioma, further evidence to support this possibility has not yet been reported. In this study, we confirmed that BTBD10 is downregulated in human glioma tissue at both the mRNA and protein levels. In addition, we detected a negative association between tumoral BTBD10 expression and the pathological grade of the tumor, which suggests that BTBD10 may act as a tumor suppressor during disease progression. In line with these clinical data, overexpression of BTBD10 inhibited the proliferation and promoted the apoptosis of U251 human glioblastoma cells *in vitro*. Furthermore, overexpression of BTBD10 increased the proportion of U251 cells at the G0/G1 phase of the cell cycle. Thus, the anti-proliferative effects of BTBD10 were likely due to the induction of G0/G1 cell-cycle arrest.

Only a limited number of published reports have examined the role of BTBD10 in cell proliferation or cancer. Previous *in vitro* studies have indicated that downregulation of BTBD10 promotes neuronal death and may contribute to the pathogenesis of amyotrophic lateral sclerosis [15,16] and brain damage after intracerebral hemorrhage [17,18]. Furthermore, overexpression of BTBD10 was observed to enhance the proliferation of pancreatic beta cells [19,20]. Additionally, the expression of BTBD10 was increased in hepatocellular carcinoma tissue, and a higher level of BTBD10 was associated with a poorer prognosis [21]. Our findings that BTBD10 was downregulated in human glioma tissue and that BTBD10 overexpression exerted anti-proliferative effects in U251 cells do not agree with these previous reports, which suggests that the functional effects of BTBD10 in glioma cells may differ from those in other cell types. Thus, BTBD10 may play a unique role in the pathogenesis of glioma.

In this study, we found that overexpression of BTBD10 in U251 cells resulted in downregulated cyclin D1 expression at the protein level. Cyclin D1 is a master regulator of cell cycle transition from the G1 phase to the S phase. Cyclin D1 forms a complex with cyclin-dependent kinase-4 (Cdk4) or Cdk6, and the resulting cyclin D1-Cdk4/6 dimer phosphorylates and inactivates retinoblastoma tumor suppressor protein. This leads to the release of transcription factor E2F, which drives the transition from the G1 phase to the S phase [22]. Cyclin D1-Cdk4 also promotes entry into the S phase by sequestering the Cdk inhibitor, p21, and consequently activating cyclin E-Cdk2 [23]. Mutation or overexpression of cyclin D1 occurs in many tumors and is thought to contribute to carcinogenesis by deregulating cell cycle progression [23,24]. Cyclin D1 expression has been shown to be associated with the pathological grade and aggressiveness of glioma, the prognosis of patients with glioma, and the response to chemotherapy [25,26,27]. The authors suggest that the induction of G0/G1 arrest and inhibition of growth caused by BTBD10 overexpression in U251 cells were likely mediated by the downregulation of cyclin D1.

The PI3K/Akt signaling pathway is a central regulator of cell cycle progression, apoptosis, and oncogenesis [28]. Phosphatase and tensin homolog (PTEN) inhibits PI3K-dependent phosphorylation and activation of Akt. PTEN is mutated or deficient in glioblastoma cells, leading to elevated Akt activity [29]. Numerous studies have demonstrated that Akt signaling plays an important role in glioma formation and progression [30]. Furthermore, small molecule inhibitors of Akt were shown to inhibit the growth and promote the apoptosis of U251 cells [31]. Several previous studies have reported that BTBD10 is an activator of Akt signaling, and it has been suggested that the binding of BTBD10 to Akt prevents the dephosphorylation of Akt by protein phosphatase 2 A [15,17,19,20,21,32]. By contrast, the present study demonstrated that p-Akt levels were decreased in U251 cells overexpressing BTBD10, implying that BTBD10 is a negative regulator of the Akt signaling pathway in glioma cells. Our findings suggest that the anti-proliferative and pro-apoptotic effects of BTBD10 overexpression were mediated via the attenuation of Akt signaling as well as the downregulation of cyclin D1. However, the detailed mechanisms by which BTBD10 downregulates cyclin D1 and p-Akt in glioma cells will require further investigation.

## 5 Conclusions

BTBD10 is downregulated in human glioma, and the expression of BTBD10 is negatively correlated with the pathological grade of the tumor. Furthermore, BTBD10 overexpression inhibits proliferation, induces G0/G1 arrest, and promotes apoptosis in human glioblastoma cells by downregulating cyclin D1 and attenuating Akt signaling. BTBD10 may be a novel therapeutic target for glioma.

## Supplementary Material

Supplementary Figure
