# Supplementary material for: BTBD10 inhibits glioma tumorigenesis by downregulating cyclin D1 and p-Akt
Source: Open Life Sci. 2022 Aug 10;17(1):907–16. doi: 10.1515/biol-2022-0103 (PMC9372705; doi:10.1515/biol-2022-0103)
Supplement: Supplementary Figure [file biol-2022-0103-sm.pdf]

Supplementary material

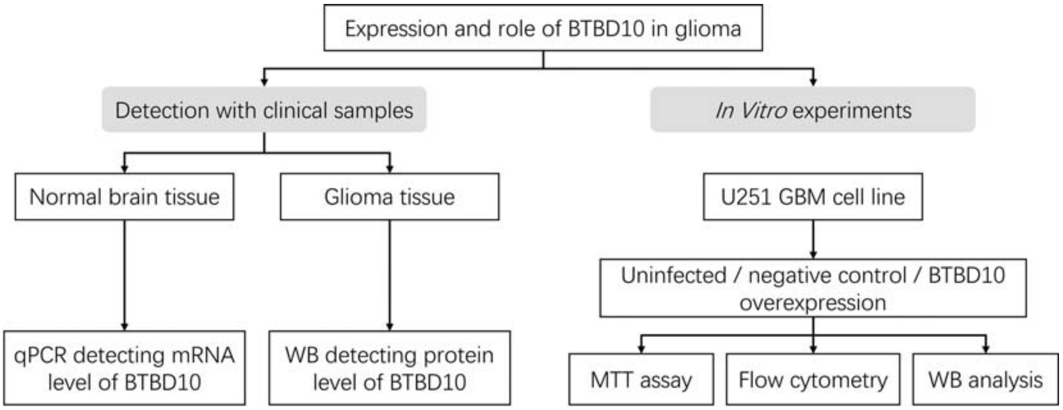

Figure S1: Schematic diagram of the study workflow.

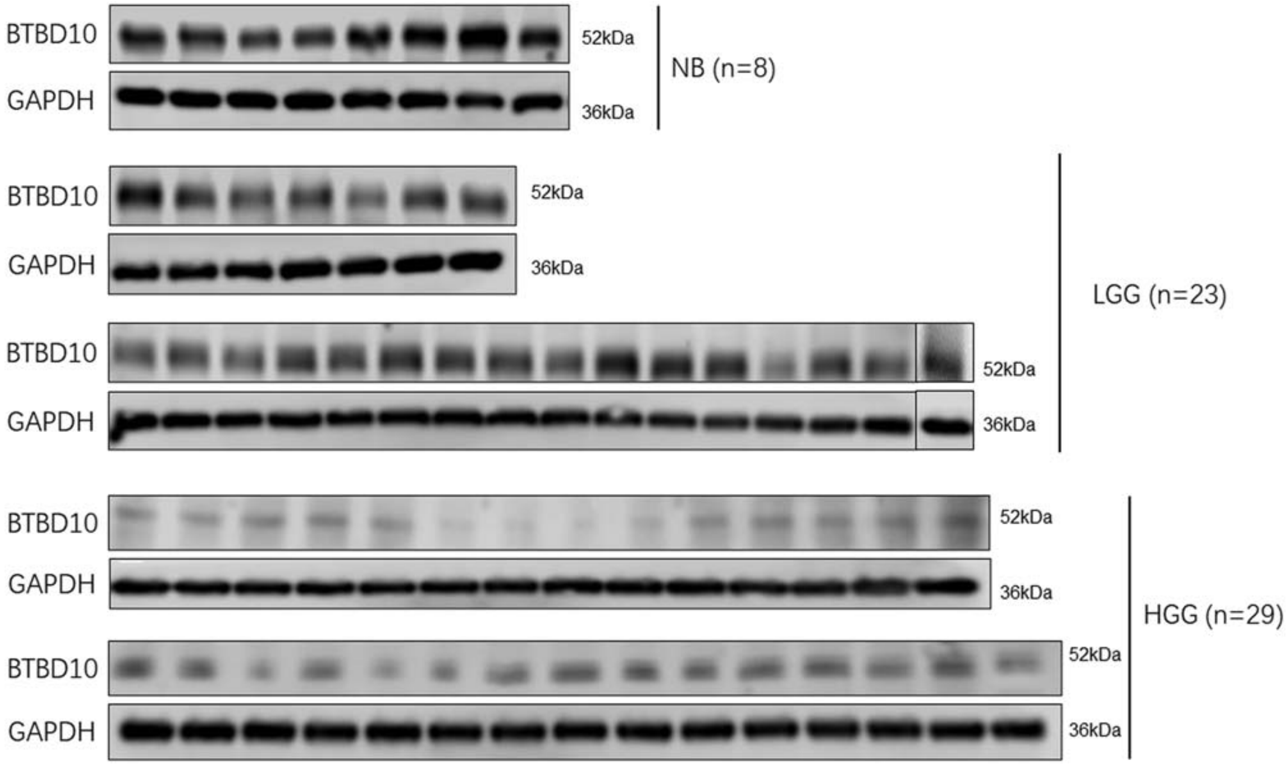

Figure S2: The protein expression of BTBD10 in all patients.

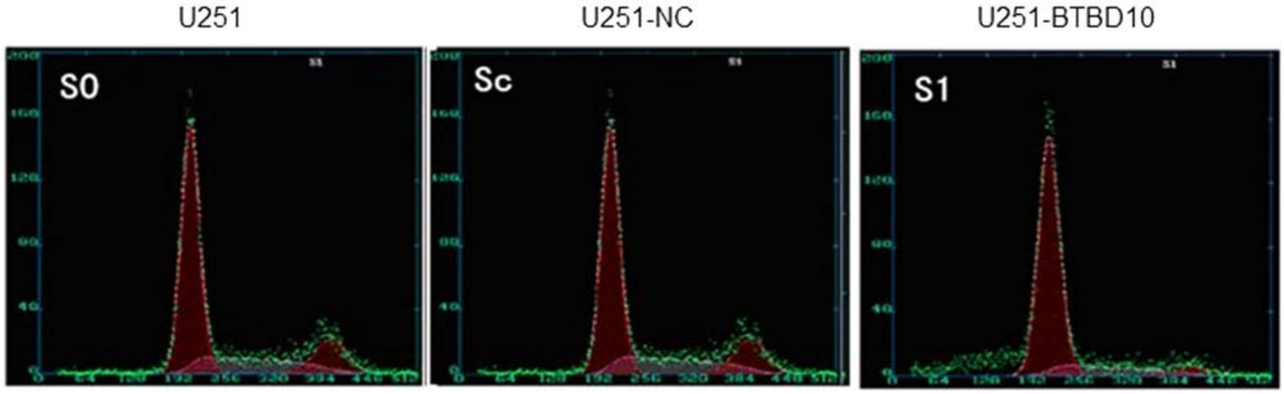

Figure S3: Original representative images for flow cytometry.
